# The parity paradox: does number of children (parity) influence breast cancer mortality across the life course?

**DOI:** 10.1186/s12885-025-14993-1

**Published:** 2025-11-11

**Authors:** Ronit Pinchas Mizrachi, Dan Bouhnik

**Affiliations:** Jerusalem College of Technology, Jerusalem, Israel

**Keywords:** Breast cancer, Parity, Mortality, Age-stratified analysis, Life course cohort study

## Abstract

While parity (number of births) has been extensively studied in relation to breast cancer risk, its age-specific impact on breast cancer mortality remains unclear. We examined how the relationship between parity and breast cancer mortality rates varies across age groups. This retrospective cohort study was based on 894,608 Israeli women born between 1940 and 1960 and followed for 31 years (1990–2020). Employing an age-stratified approach (30–49, 50–64, and 65–80 years), we used Cox regression to examine the associations between number of children (0, 1–2, and ≥ 3) and breast cancer mortality rates, adjusting for sociodemographic variables and calendar year of entry into each age group. Amongst the 30–49 bracket, those with 1–2 children (hazard ratio [HR] = 1.656; 99% confidence interval [CI] 1.349–2.033) or ≥ 3 children (HR = 1.551; 99% CI 1.271–1.893) had a greater breast cancer mortality risk than childless women. In the 50–64 bracket, these differences disappeared after adjustment (HR = 1.071; 99% CI 0.949–1.209 and HR = 0.935; 99% CI 0.830–1.054, respectively). In the 65–80 bracket, those with 1–2 children continued to exhibit an elevated risk (HR = 1.237; 99% CI 1.045–1.466), no significant difference existing between women with ≥ 3 children and childless women (HR = 0.989; 99% CI 0.834–1.173). An age-dependent relationship thus obtained between parity and breast cancer mortality. These findings highlight the need for age-tailored approaches to breast cancer risk assessment and prevention.

**Supplementary Information:**

The online version contains supplementary material available at 10.1186/s12885-025-14993-1.

## Background

The most commonly diagnosed malignancy amongst women worldwide, breast cancer remains a leading cause of cancer-related mortality. Despite advances in screening and treatment methods, projections indicate that by 2050, breast cancer incidence and mortality rates will increase considerably. This forecast underscores the urgent need for further investigation into modifiable risk factors [1].

Breast cancer risk is influenced by genetic, environmental, and lifestyle factors. While obesity increases the risk of breast cancer in menopausal and postmenopausal women, it may offer protection to premenopausal women. Regular physical activity and weight management provide protective benefits [2]. Women who are postmenopausal face increased risk due to changes in estrogen metabolism, body composition, and immune function [3].

Reproductive history also affects breast cancer risk. A meta-analysis evinced that early menstruation (age < 13 years), nulliparity, and hormonal contraceptive use are predictors of breast cancer in women of reproductive age [4]. Studies of infertility and breast cancer risk remain inconclusive, a modest association with postmenopausal breast cancer existing, particularly amongst women with early infertility [5].

## Pregnancy-associated breast cancer

Pregnancy-associated breast cancer occurs during pregnancy or after delivery and is characterized by more aggressive tumors, delayed diagnosis, and worse outcomes. Post-delivery diagnosis increases the risk of metastasis, likely due to immune suppression enabling tumor spread [6]. Pregnancy can promote short-term breast cancer risk while reducing lifetime risk [7].

### Breastfeeding and hormonal contraceptives

Breastfeeding provides considerable protection against breast cancer, particularly for hormone receptor-negative subtypes. It lowers estrogen levels and helps eliminate damaged epithelial cells, longer breastfeeding duration providing greater protection [8]. Conversely, hormonal contraceptives increase breast cancer risk through sustained estrogen–progesterone stimulation of breast cells. This risk decreases following discontinuation of these contraceptives, however [4].

## Risk factors during and after menopause

The menopausal transition represents a critical period for breast cancer risk. Women experiencing menopause at a later age remain exposed to endogenous estrogen for longer, thus running a greater risk of hormone receptor-positive breast cancers [9]. Hormone replacement therapy (HRT), especially combined estrogen-progesterone therapy, is associated with increased breast cancer risk, in particular with long-term use [10, 11].

High-density breast tissue complicates early tumor detection, postmenopausal genetic changes potentially contributing to cancer development [12, 13]. Obesity increases the risk of breast cancer in women who are menopausal or postmenopausal, highlighting complex hormonal interactions across life stages [2].

Postmenopausal women are at greater risk of breast cancer due to changes in estrogen metabolism, inflammation, and immune function. Inflammation-driven tumor promotion in aging breast tissue also serves as a contributing factor [3]. Estrogen receptor-positive cancers are particularly influenced by endogenous hormones, the risk decreasing shortly after menopause due to reduced estrogen levels [14].

The obesity many women experience after multiple births [15] may also increase breast cancer risk and reduce survival rates in postmenopausal women [3, 16].

## Number of children as a risk or protective factor

Parity (number of births) has been identified as a key modifier of breast cancer risk. Its impact on long-term survival outcomes nonetheless remains unclear [9]. A study of 385,816 married women in Norway found a significant decrease in breast cancer incidence with each additional pregnancy, a 10.5% reduction per additional child occurring in high parity cases of [17].

Regarding the relationship between parity and breast cancer survival, a study of 1,485 African women diagnosed with breast cancer found that each pregnancy increased mortality risk by 5%. Premenopausal women who gave birth within the three years prior to their diagnosis exhibited a 52% lower survival rate [18]. Another study found that younger women (aged < 40 years)—particularly in hormone receptor-positive subtypes—run a higher risk of breast cancer mortality [19].

Early studies found that nulliparous women were at higher risk of developing breast cancer than those who had their first child before the age of 20. This suggests that Lifetime risk can be reduced by up to 50% [20]. More recent research indicates that the protective effect of parity varies across breast cancer subtypes, however, no consistent association obtaining between parity and HER2-positive or triple-negative breast cancer [21, 22].

## Fertility in Israel and identified risk factors

Israelis—particularly those within the Arab and ultra-Orthodox communities—have a relatively higher fertility rate than other Western women, hormonal contraceptive use being relatively uncommon and fertility treatments in wide use [23, 24].

Previous studies conducted in this cohort reported notable disparities in breast cancer mortality rates, Jewish women experiencing higher mortality rates than Muslim women [25], non-Haredi Jewish women than Haredi Jewish women [26], and urban than rural women [27].

## Study objectives

The study examined the age-dependent relationship between parity and breast cancer mortality in a large cohort of 894,608 Israeli women followed for 31 years. Although parity is well established as a factor influencing breast cancer incidence, its potential impact on mortality may reflect both biological mechanisms (e.g., pregnancy-associated tumor aggressiveness) and social factors, such as delayed diagnosis due to caregiving responsibilities. Adopting an innovative age-stratified approach looking three distinct life stages, it sought to determine whether the association between number of children and breast cancer mortality varies across a woman’s life course and persists after adjusting for sociodemographic variables.

We selected potential confounding variables—ethnicity, ultra-Orthodox identity, residential locality size, socioeconomic status, and country of origin—on the basis of previous findings from this cohort that demonstrated their importance in breast cancer mortality patterns.

## Methods

### Study design and population

The cohort comprised 894,608 Israeli women born between 1940 and 1960, representing the general populace rather than breast cancer patients, whom we followed for 31 years (1 January, 1990–31 December, 2020). The women were aged between 30 and 50 at entry and 60 and 80 at the termination. We employed a unique age-stratified approach to examine breast cancer mortality rates, the individual-level data being obtained from diverse official sources—the Population Authority, Tax Authority, Education Ministry, Central Bureau of Statistics (CBS), and Ministry of Health. Each individual was assigned a fictitious ID number used consistently across all databases. This enabled comprehensive data integration while maintaining confidentiality.

The study received ethical approval from the Tax Authority, Population Registry, and CBS ethics committees, all the data processing and analysis being conducted in the CBS research room after obtaining ethical approval.

It adopted a retrospective cohort design to track long-term breast cancer mortality amongst a defined national population. Parity—number of live-born children—and other sociodemographic characteristics were obtained at baseline (1990) from linked administrative datasets provided by the Population Registry, CBS, Ministry of Health, and other government sources.

One of the study’s unique features was following a specific cohort over three decades in order to observe the evolving relationship between parity and breast cancer mortality. Rather than treating age as a time-varying covariate, we stratified follow-up into three distinct life-course periods—30–49, 50–64, and 65–80. This approach enabled us to capture age-specific patterns while controlling for confounders within each age band.

Breast cancer-specific mortality was ascertained on the basis of national death records. Cox proportional hazards models were used separately within each age group, adjusting for birth cohort, education, population group, country of origin, and locality type. The proportional hazards assumption tested via Schoenfeld residuals was found to be adequately met for all models.

### Study variables

The sociodemographic variables collected from the Population Registry covered sex, year of birth, religion, ethnicity, size of locality of residence, and country of origin—all of which were constructed as potential confounding variables.

Education level was divided into three groups on the basis of data from the Education Registry (managed by the CBS): high (≥ 13 years of education), intermediate (8–12 years of education), and low (up to 8 years of education). Individuals with missing education data were classified as low as per standard CBS procedure.

Ethno-religious grouping was divided into Haredi Jewish, non-Haredi Jewish, and Arab and others. Following CBS conventions, all non-Jewish minorities—including Arabs and smaller groups such as Druze and Circassians—were combined into a single category. This classification ensures consistency with national data and statistical stability.

All the variables were treated as potential confounders on the basis of a literature review and previous cohort findings.

The primary exposure variable was number of children—no children (childless), 1–2 children, and ≥ 3 children. Parity was treated a categorical variable in all the models, nulliparous women serving as the reference group.

The outcome variable was breast cancer mortality, determined on the basis of Ministry of Health death certificates.

### Method used for age-based follow-up period division

A key methodological feature of this study was the use of an age-stratified analysis approach within a single cohort. To examine age-specific impacts on breast cancer mortality rates, each woman contributed to relevant age groups based on the number of years Lived within that specific interval. Only survivors from each age group who remained in Israel were included in subsequent age groups. Mortality rates were calculated based on the number of person-years. In the 30–49 years age range, 866,160 women participated, contributing 9,780,823 person-years; in the 50–64 years age range, 857,104 women participated, contributing 12,179,461 person-years; and in the 65–80 years age range, 752,958 women participated, contributing 3,853,657 person-years.

This approach created a situation where women born in 1940 who entered the cohort at age 50 years did not contribute to the 30–49 years age range, while women born in 1960 who were aged 60 years at the end of follow up did not contribute to the 65–80 years age range.

To prevent bias from improvements in breast cancer survival rates over time, statistical models across age ranges were adjusted for the calendar year of entry into each specific age range.

### Statistical analysis

We examined the distribution of various baseline characteristics, including age at study entry, level of education, ethno-religious group (non-Haredi Jewish, Haredi Jewish, or Arab), country of origin, and size of locality of residence. Chi-square tests were used to compare the frequency distributions of the categorical variables and Analysis of Variance (ANOVA) for age comparisons across the parity groups.

Breast cancer mortality rates per 10,000 women over the entire study period were stratified by education, ethno-religious group, country of origin, and size of locality of residence. Adjusted hazard ratios (AHRs) for breast cancer mortality were calculated, adjusting for age at the time of study entry. Employing Cox regression, adjusted Kaplan-Meier analysis, and controlling for entry age, we calculated the relationship between the study variables and breast cancer mortality. Effect estimates are presented as hazard ratios (HRs) with 99% confidence intervals (CIs).

We examined breast cancer mortality rates per 10,000 women and 100,000 person-years by number of children and age-based follow-up period (30–49, 50–64, and 65–80 years) for the overall population throughout the entire follow-up period. Employing Cox regression and adjusted Kaplan-Meier analysis and controlling for age at the time of study entry, we evaluated the relationship between number of children and breast cancer mortality.

We constructed additional Cox regression models and adjusted Kaplan-Meier curves to evaluate the relationship between number of children and breast cancer mortality while controlling for age at study entry/entry year in the age-based follow-up period, level of education, ethno-religious group, country of origin, and size of locality of residence. The models were compared via Likelihood ratio tests. Effect estimates are presented as HRs and 99% CIs. All the analyses were conducted on the total population and in accordance with age-based follow-up periods.

Women who emigrated from Israel during the study period only contributed to the number at risk in survival analyses until the date of their departure.

All statistical analyses were performed via SPSS software (version 29).

## Results

### Study design and population

The cohort study followed 894,608 Israeli women born between 1940 and 1960 over a 31-year period (1990–2020). After analysis, the entire cohort was divided into age-based follow-up groups. Only those who entered each age period while still alive were included in the respective analyses, thereby ensuring accurate longitudinal mortality tracking.

### Distribution of study variables

The mean age at study entry was highest amongst childless women (39.95 years; SD = 5.65), followed by those with 1–2 children (38.63 years; SD = 5.42), and ≥ 3 children (38.70 years; SD = 5.95; *P* < 0.001). Significant differences were observed in ethno-religious composition, level of education, country of origin, and size of locality of residence, childless women being more educated and more represented amongst the European- and American-born groups (Table 1).

**Table 1:** Sociodemographic characteristics of the study population by number of children (*N* = 894,608 women)

Variable		Number of children			*P*-value
		No children	1–2 children	≥ 3 children	
	Number of women (%)	***n*** **= 183**,**500** (20.5%)	***n*** **= 284**,**841** (31.8%)	***n*** **= 426**,**627** (47.6%)	
Mean age, years, at the beginning of the study (SD)		39.95 (5.65)	38.63(5.42)	38.70 (5.95)	< 0.001
Ethno-religious group	Non-Haredi Jewish	86.2	95.1	75.6	< 0.001
	Haredi Jewish	0.9	1.6	6.2	
	Arab	12.9	3.3	18.2	
Education (years)	0–8	50.9	21.4	27.2	< 0.001
	9–12	15.2	28.8	39.9	
	≥ 13	33.9	49.8	32.8	
Country of origin	Europe/America	55.7	62.6	23.2	< 0.001
	Asia/Africa	20.0	18.6	38.1	
	Israel	24.3	18.8	38.5	
Size of locality of residence	Small	15.7	10.8	24.4	< 0.001
	Large	84.3	89.2	75.6	

*SD* Standard deviation

### Breast cancer mortality by number of children in the entire follow-up period

The overall breast cancer mortality rate during the study period was 86.61 per 10,000 women. A significant association obtained between number of children and breast cancer mortality risk (*P* < 0.001), women with 1–2 children or ≥ 3 children running a higher risk of breast cancer mortality than childless women (HR = 1.375; 99% CI 1.270–1.487 and HR = 1.213; 99% CI 1.125–1.308 respectively) (Table 2).

**Table 2:** Breast cancer mortality rates by sociodemographic characteristics among the total population (*N* = 894,608 women)

		Mortality rate per 10,000 during the study period	Age-adjusted hazard ratio (99% CI)	*P*-value
Number of children	0	86.61	1.00	
	1–2	111.75	1.375 (1.270–1.487)	< 0.001
	≥ 3	99.47	1.213 (1.125–1.308)	< 0.001
Ethno-religious group	Non-Haredi Jewish	106.49	1.00	
	Haredi Jewish	81.11	0.812 (0.690–0.956)	< 0.001
	Arab	85.40	0.871 (0.793–0.956)	< 0.001
Education (years)	0–8	85.51	1.00	
	9–12	110.21	1.483 (1.382–1.592)	< 0.001
	≥ 13	107.25	1.402 (1.310–1.501)	< 0.001
Country of origin	Asia/Africa	96.99	1.00	
	Israel	105.25	1.071 (0.997–1.143)	0.013
	Europe/America	107.60	1.109 (1.031–1.192)	< 0.001
Size of locality of residence	Small	84.30	1.00	
	Large	105.10	1.212 (1.125–1.306)	< 0.001

*CI* Confidence interval

After adjustment for age at study entry, level of education, ethno-religious group, country of origin, and size of locality of residence, women with 1–2 children continued to exhibit a higher risk of breast cancer mortality than childless women (HR = 1.216; 99% CI 1.117–1.324), no significant difference obtaining between women with ≥ 3 children and childless women (HR = 1.056; 99% CI 0.970–1.149).

### Breast cancer mortality by number of children in our age-based follow-up analysis

#### 30–49 bracket follow-up period

Those with 1–2 children and ≥ 3 children exhibited higher breast cancer mortality rates than childless women (HR = 1.195; 99% CI 1.092–1.307 and HR = 1.140; 99% CI 1.140–1.166 respectively) (Table 3).

**Table 3:** Breast cancer mortality rates by number of children in the total population and by age-based follow-up groups (30–49, 50–64, and 65–80 years)

	Number of children	Total population	30–49 years age-based follow-up group	50–64 years age-based follow-up group	65–80 years age-based follow-up group
Number of women	0	183,500	175,033	173,100	136,152
	1–2	284,841	277,462	274,290	234,494
	≥ 3	426,267	413,665	409,714	382,312
Cumulative follow-up person-years	0	5,150,465	1,817,985	2,480,704	851,776
	1–2	8,243,729	3,196,267	3,888,177	1,159,285
	≥ 3	12,419,747	4,766,571	5,810, 580	1,842,596
Breast cancer mortality rate per 10,000 in the follow-up period	0	86.61	14.00	48.18	31.07
	1–2	111.75	24.36	58.55	32.11
	≥ 3	99.47	22.51	52.57	25.21
Breast cancer mortality rate per 100,000 person-years	0	29.29	13.48	33.62	49.66
	1–2	36.81	21.15	41.30	64.95
	≥ 3	32.75	19.53	37.07	52.32
Aged-adjusted hazard ratio for breast cancer mortality	0	1.00	1.00	1.00	1.00
	1–2	1.375 (1.270–1.487)^a^	1.195 (1.092–1.307)^b^	1.273 (1.140–1.421)^b^	1.351 (1.155–1.581)^b^
	≥ 3	1.213 (1.125–1.308)^a^	1.140 (1.114–1.166)^b^	1.141 (1.027–1.267)^b^	1.082 (0.931–1.258)^b^

^a^Adjusted for age at the beginning of the study

^b^Adjusted for entry year in the age-based follow-up period

The association strengthened after adjustment for entry year, level of education, ethno-religious group, country of origin, and size of locality of residence, women with 1–2 children (HR = 1.656; 99% CI 1.349–2.033) and those with ≥ 3 children (HR = 1.551; 99% CI 1.271–1.893) continuing to exhibit higher breast cancer mortality rates than childless women. No significant differences in mortality were observed between women with 1–2 children and those with ≥ 3 children (Table 4).

**Table 4:** Results of multivariable Cox models for predicting breast cancer mortality rates by number of children and sociodemographic variables (hazard ratio) in the total population and by age-based follow-up groups (30–49, 50–64, and 65–80 years)

*N*		^a^Model A total population	Model B^b^ 30–49 years age-based follow-up group	^b^Model C 50–64 years age-based follow-up group	^b^Model D 65–80 years age-based follow-up group
		894,608	866,160	857,104	752,958
Number of children	0	1.00	1.00	1.00	1.00
	1–2	1.216 (1.117–1.324)	1.656 (1.349–2.033)	1.071 (0.949–1.209)	1.237 (1.045–1.466)
	≥ 3	1.056 (0.970–1.149)	1.551 (1.271–1.893)	0.935 (0.830–1.054)	0.989 (0.834–1.173)
Ethno-religious group	Non-Haredi Jewish	1.00	1.00	1.00	1.00
	Haredi Jewish	0.776 (0.658–0.915)	0.720 (0.623–0.865)	0.873 (0.703–1.084)	0.711 (0.54–0.935)
	Arab	0.852 (0.715–0.911)	0.784 (0.601–0.962)	1.079 (0.922–1.263)	0.825 (0.648–1.051)
Education (years)	0–8	1.00	1.00	1.00	1.00
	9–12	1.471 (1.361–1.589)	1.254 (1/058 − 1/487)	1.558 (1.392–1.745)	1.478 (1.263–1.665)
	≥ 13	1.388 (1.283–1.501)	0.965 (0.886–1.051)	1.598 (1.428–1.788)	1.422 (1.263–1.729)
Country of origin	Asia/Africa	1.00	1.00	1.00	1.00
	Israel	0.994 (0.923–1.072)	1.028 (0.873–1.210)	0.950 (0.855–1.055)	0.977 (0.836–1.142)
	Europe/America	1.136 (1.050–1.230)	1.254 (1.059–1.487)	1.088 (0.974–1.215)	1.019 (0.963–1.203)
Size of locality of residence	Small	1.00	1.00	1.00	1.00
	Large	1.205 (1.113–1.304)	1.298 (1.094–1.541)	1.123 (1.007–1.251)	1.278 (1.077–1.516)

^a^Adjusted for age at the beginning of study

^b^Adjusted for entry year in the age-based follow-up period

#### 50–64 bracket follow-up period

Those with 1–2 children and ≥ 3 children exhibited higher breast cancer mortality rates than childless women (HR = 1.273; 99% CI 1.140–1.421 and HR = 1.141; 99% CI 1.027–1.267 respectively).

Women with 1–2 children (HR = 1.071; 99% CI 0.949–1.209) or ≥ 3 children (HR = 0.935; 99% CI 0.830–1.054) exhibited no significant difference in breast cancer mortality rates in comparison with childless women after adjustment for entry year, level of education, ethno-religious group, country of origin, and size of locality of residence, howeve

#### 65–80 bracket follow-up period

Those with 1–2 children exhibited higher breast cancer mortality rates than childless women (HR = 1.351; 99% CI 1.155–1.581), no significant differences obtaining between women with ≥ 3 children and childless women (HR = 1.082; 99% CI 0.931–1.258). Women with 1–2 children continued to exhibit higher breast cancer mortality rates than childless women after adjustment for entry year, level of education, ethno-religious group, country of origin, and size of locality of residence (HR = 1.237; 99% CI 1.045–1.466), no significant differences existing between women with ≥ 3 children and childless women (HR = 0.989; 99% CI 0.834–1.173). 

### Summary of findings

**Fig. 1:**
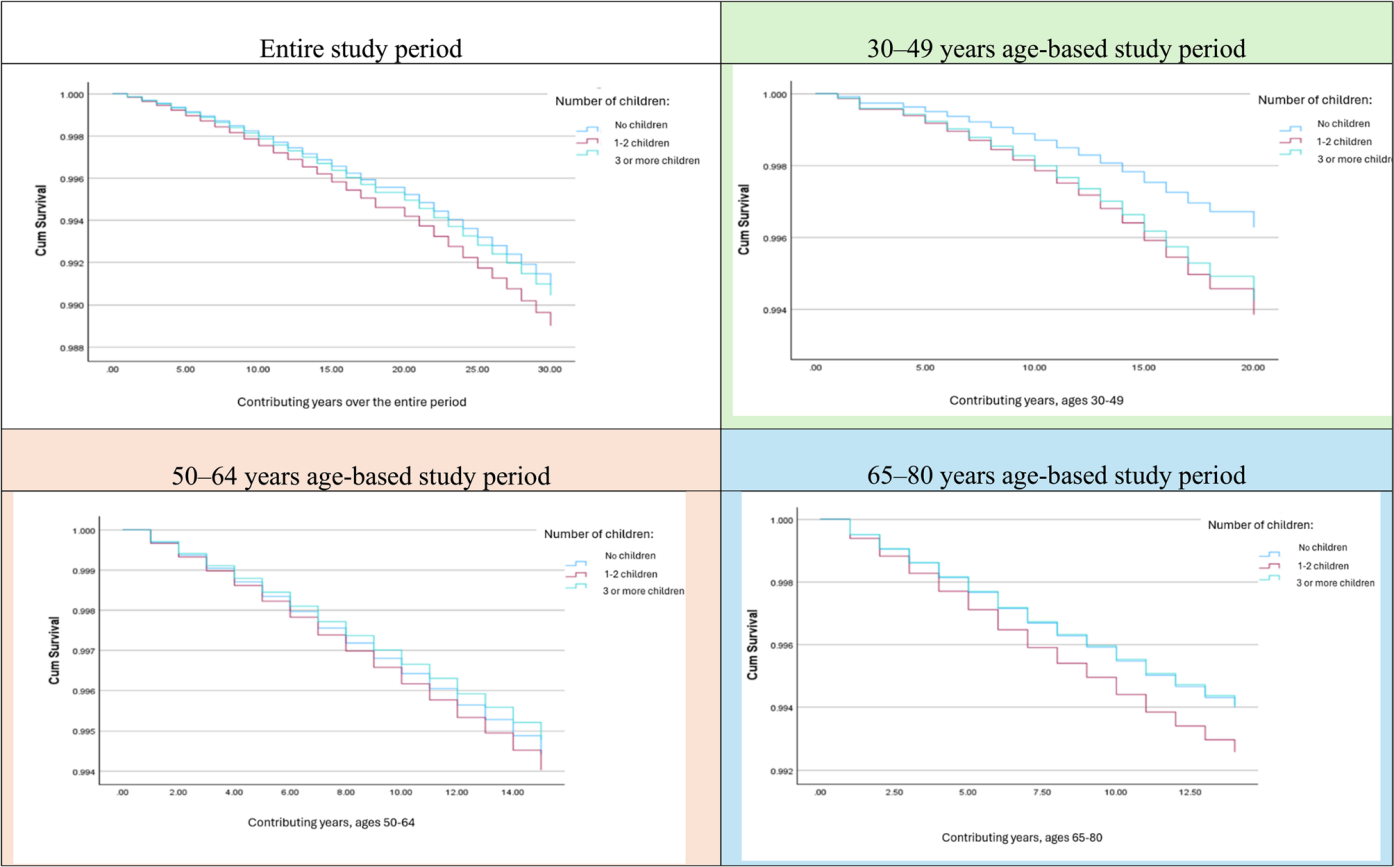
Analysis of breast cancer mortality rates by number of children, adjusted for age at the beginning of the follow-up period, level of education, ethno-religious group, country of origin, and size of locality of residence in the total population and by age-based follow-up groups (30–49, 50–64, and 65–80 years)

The study revealed an age-dependent relationship between parity and breast cancer mortality rates, motherhood being associated with increased mortality risk due to breast cancer in younger women (30–49 years), weaker risk in middle-aged women (50–64 years), and a varied risk in older women (65–80 years). These findings highlight the need for age-stratified approaches to breast cancer risk assessment and prevention (Fig. 1).

## Discussion

In this study, we employed a unique methodological approach involving an age-stratified analysis within a single cohort, enabling precise calculations of mortality rates. The strengths of our study include its complete population coverage, extended follow-up period, adjustment for sociodemographic variables, and implementation within a universal healthcare system.

Biological mechanisms may also help explain the observed patterns. Elevated levels of estrogen, progesterone, and growth factors stimulate mammary cell proliferation during pregnancy, thus potentially increasing susceptibility to oncogenic transformation [20]. Immune tolerance mechanisms that protect the fetus may allow tumor cells to evade detection, extensive post-lactation tissue remodeling also sharing features with pro-oncogenic environments. Pregnancy-associated breast cancer (PABC) further exhibits distinct gene expression patterns and is often more aggressive in nature. These mechanisms may help clarify the elevated breast cancer mortality observed amongst younger parous women.

Our findings revealed a significant age-dependent relationship between parity and breast cancer mortality rates. While previous studies indicate that childbirth protects against breast cancer, ours suggest a more complex association, particularly with respect to mortality rates. While women who give birth at a young age or have multiple children tend to run a lower risk of developing breast cancer than childless women, this effect pertains to disease incidence rather than mortality risk [20, 22]. Differences in breast cancer subtypes must also be considered, nulliparous women being at increased risk of certain subtypes of breast cancer such as luminal A and B but no consistent association obtaining for HER2-positive or triple-negative cancers [22, 28].

Women in the 30–49 bracket with children exhibited a higher breast cancer mortality rate than childless women, even after adjustment for confounders. This may be due to pregnancy-associated breast cancer, which tends to be more aggressive and diagnosed in its later stages [4, 6]. Mothers may also have less time for self-care and attending breast cancer screening appointments. Previous studies evince that women with higher parity may attend fewer mammography screenings, possibly due to their greater caregiving responsibilities and limited time or financial resources. Stimpson et al. [29] report that only 42% of women with two or more children underwent a mammography compared to 55% of childless women, for example [30]. These preventive-care disparities may partially explain the increased breast cancer mortality risk observed among women with children, particularly in the younger age groups.

The absence of any significant difference between women with 1–2 children and those with ≥ 3 children suggests the presence of competing factors—increased risk from pregnancy-related hormonal changes vs. potential long-term hormonal protection from multiple pregnancies [7].

The association between parity and breast cancer mortality rates weakened in the 50–64 bracket, disappearing altogether after adjustment for confounders. This finding may be due to menopause-related hormonal changes that affect all women similarly, regardless of their reproductive history. Widespread mammography screening in Israel likely further contributes to improved early detection and survival in this age bracket [31].

Only women with 1–2 children had a higher breast cancer mortality risk than childless women in the 65–80 bracket, no significant difference obtaining between those with ≥ 3 children and childless women. This finding is consistent with previous research that indicates that high parity may provide long-term protection, potentially due to cumulative hormonal exposure and epigenetic changes that influence cancer risk [28]. The biological response to multiple pregnancies and decline in immune function in older age may further impact breast cancer outcomes.

The unexpected finding that childlessness serves as a protective factor may be unique to Israel, where fertility rates high and society places a premium on parenthood [26]. In contrast to Western countries, in which women commonly use contraceptives or terminate pregnancy, Israeli childlessness is often involuntary [24, 32]. Fertility treatments are also widespread in Israel, women with 1–2 children likely to undergo such treatments and thereby potentially increasing their risk of breast cancer morbidity and mortality [23].

Obesity—more prevalent amongst women who have given birth multiple times—is linked to both increased breast cancer risk and poorer survival outcomes, potentially narrowing the gap between nulliparous and parous women [3, 15, 16].

Despite its strengths, the study suffers from a number of limitations. Resting on administrative data, it lacks details regarding morbidity, breastfeeding, age at first birth, fertility treatments, contraceptive use, adherence to breast cancer screening programs (e.g. mammography), cancer stage at diagnosis, and behavioral risk factors such as smoking and physical inactivity. Pregnancy numbers are accurately measurable, in particular amongst immigrants—for whom data regarding deceased children may be incomplete or unavailable due to historical population-registration limitations. Methodologically, the follow-up periods differed across age groups (30–49: 1990–2009; 50–64: 1990–2020; 65–80: 2005–2020). Although we adjusted for entry year, this limitation should be considered. Finally, Israel’s unique childbirth patterns and demographic characteristics may limit the generalizability of these findings to other populations [23, 33]. Further research in diverse settings is thus required before the applicability of these results can be properly assessed.

## Conclusions

The study demonstrates that childbearing impacts breast cancer mortality risk variantly across the lifespan, both biological and social factors shaping complex age-specific patterns. Motherhood increased breast cancer mortality risk in younger women (30–49) compared with in childless women—an effect that weakened in middle age (50–64) and partially persisted in older women (65–80 years) only amongst those with 1–2 children.

The age-stratified approach challenges traditional views of parity as protecting against breast cancer mortality by revealing an age-dependent relationship between parity and breast cancer mortality that conventional analyses might overlook. In light of the study’s observational nature, causal interpretations must be adopted cautiously. Residual confounding by unmeasured clinical and behavioral variables cannot be ruled out.

Future research should explore the underlying mechanisms of these associations through prospective cohort studies that rest on detailed reproductive histories, parity-cancer subtype relationships, and cross-cultural analyses. These insights should yield more nuanced, age-specific risk assessment strategies for breast cancer, tailored to reproductive history and life stage.

## Supplementary Information

Supplementary Material 1.

Supplementary Material 2.

## Data Availability

The data used in this study are available from the Israeli Central Bureau of Statistics, subject to reasonable request and approval by the CBS Research Room.
