# Correction: The parity paradox: does number of children (parity) influence breast cancer mortality across the life course?

**DOI:** 10.1186/s12885-025-15369-1

**Published:** 2025-11-27

**Authors:** Ronit Pinchas-Mizrachi, Dan Bouhnik

**Affiliations:** Jerusalem College of Technology, Jerusalem, Israel

**Correction: BMC Cancer 25**,** 1750 (2025)**

**https://doi.org/10.1186/s12885-025-14993-1**

Following the publication of the original article [1], the authors reported an error in the corresponding author’s family name. It was incorrectly captured as “Mizrachi” instead of “Pinchas-Mizrachi.”
