# Supplementary material for: The parity paradox: does number of children (parity) influence breast cancer mortality across the life course?
Source: BMC Cancer. 2025 Nov 11;25:1750. doi: 10.1186/s12885-025-14993-1 (PMC12606814; doi:10.1186/s12885-025-14993-1)
Supplement: Supplementary file 1 — Supplementary Material 1. [file 12885_2025_14993_MOESM1_ESM.docx]

*Table 1: Sociodemographic characteristics of the study population by number of children* *(N = 894,608 women)*

|  | **Number of children** | | |  | **Variable** |
| --- | --- | --- | --- | --- | --- |
| ***P*-value** | ≥ **3 children** | **1–2 children** | **No children** |  |  |
|  | ***n* = 426,627**  **(47.6%)** | ***n* = 284,841**  **(31.8%)** | ***n* = 183,500**  **(20.5%)** | **Number of women**  **(%)** |  |
| < 0.001 | 38.70 (5.95) | 38.63(5.42) | 39.95 (5.65) |  | Mean age, years, at the beginning of the study (SD) |
| < 0.001 | 75.6 | 95.1 | 86.2 | Non-Haredi Jewish | Ethno-religious group |
|  | 6.2 | 1.6 | 0.9 | Haredi Jewish |  |
|  | 18.2 | 3.3 | 12.9 | Arab and other |  |
| < 0.001 | 27.2 | 21.4 | 50.9 | 0–8 | Education (years) |
|  | 39.9 | 28.8 | 15.2 | 9–12 |  |
|  | 32.8 | 49.8 | 33.9 | ≥ 13 |  |
| < 0.001 | 23.2 | 62.6 | 55.7 | Europe/America | Country of origin |
|  | 38.1 | 18.6 | 20.0 | Asia/Africa |  |
|  | 38.5 | 18.8 | 24.3 | Israel |  |
| < 0.001 | 24.4 | 10.8 | 15.7 | Small | Size of locality of residence |
|  | 75.6 | 89.2 | 84.3 | Large |  |

SD = standard deviation

*Notes*

Parity was categorized as 0, 1–2, and ≥3 children. Variables include age at baseline, ethno-religious group, education, country of origin, and locality size
Arabs and others = all non-Jewish minorities per CBS standards

P-values reflect group differences on the basis of chi-square tests (categorical variables) and ANOVA (continuous variables)

*Table 2: Breast cancer mortality rates by sociodemographic characteristics amongst the total population (N = 894,608 women)*

| *P*-value | Age-adjusted hazard ratio  (99% CI) | Mortality rate per 10,000  during the study period |  |  |
| --- | --- | --- | --- | --- |
|  | **1.00** | **86.61** | **0** | **Number of children** |
| < 0.001 | **1.375**  **(1.270–1.487)** | **111.75** | **1–2** |  |
| < 0.001 | **1.213**  **(1.125–1.308)** | **99.47** | **≥ 3** |  |
|  | 1.00 | 106.49 | Non-Haredi Jewish | Ethno-religious group |
| < 0.001 | 0.812  (0.690–0.956) | 81.11 | Haredi Jewish |  |
| < 0.001 | 0.871  (0.793–0.956) | 85.40 | Arab and other* |  |
|  | 1.00 | 85.51 | 0–8 | Education (years) |
| < 0.001 | 1.483  (1.382–1.592) | 110.21 | 9–12 |  |
| < 0.001 | 1.402  (1.310–1.501) | 107.25 | ≥ 13 |  |
|  | 1.00 | 96.99 | Asia/Africa | Country of origin |
| 0.013 | 1.071  (0.997–1.143) | 105.25 | Israel |  |
| < 0.001 | 1.109  (1.031–1.192) | 107.60 | Europe/America |  |
|  | 1.00 | 84.30 | Small | Size of locality of residence |
| < 0.001 | 1.212  (1.125–1.306) | 105.10 | Large |  |

*Notes*

Breast cancer mortality rates (per 10,000 person-years) were calculated separately for three age groups—30–49, 50–64, and 65–80 years.

Parity was categorized as 0, 1–2, and ≥3 children. Rates are age-specific and reflect crude mortality within each follow-up age band

“Arabs and others” = all non-Jewish minorities per CBS standards

CI = confidence interval

Hazard ratios were only adjusted for age

*Table 3: Breast* cancer *mortality rates by number of children in the total population and age-based follow-up groups (30–49, 50–64, and 65–80 years)*

| **65–80 bracket age-based follow-up group** | **50–64 bracket age-based follow-up group** | **30**–**49 bracket age-based follow-up group** | **Total population** | **Number of children** |  |
| --- | --- | --- | --- | --- | --- |
| 136,152 | 173,100 | 175,033 | 183,500 | 0 | Number of women |
| 234,494 | 274,290 | 277,462 | 284,841 | 1–2 |  |
| 382,312 | 409,714 | 413,665 | 426,267 | ≥ 3 |  |
| 851,776 | 2,480,704 | 1,817,985 | 5,150,465 | 0 | Cumulative follow-up person-years |
| 1,159,285 | 3,888,177 | 3,196,267 | 8,243,729 | 1–2 |  |
| 1,842,596 | 5,810, 580 | 4,766,571 | 12,419,747 | ≥ 3 |  |
| 31.07 | 48.18 | 14.00 | 86.61 | 0 | Breast cancer mortality rate per 10,000 in the follow-up period |
| 32.11 | 58.55 | 24.36 | 111.75 | 1–2 |  |
| 25.21 | 52.57 | 22.51 | 99.47 | ≥ 3 |  |
| 49.66 | 33.62 | 13.48 | 29.29 | 0 | Breast cancer mortality rate per 100,000 person-years |
| 64.95 | 41.30 | 21.15 | 36.81 | 1–2 |  |
| 52.32 | 37.07 | 19.53 | 32.75 | ≥ 3 |  |
| 1.00 | 1.00 | 1.00 | 1.00 | 0 | Aged-adjusted hazard ratio for breast cancer mortality |
| 1.351  (1.155–1.581)** | 1.273  (1.140–1.421)** | 1.195  (1.092–1.307)** | 1.375  (1.270–1.487)* | 1–2 |  |
| 1.082  (0.931–1.258)** | 1.141  (1.027–1.267)** | 1.140  (1.114–1.166)** | 1.213  (1.125–1.308)* | ≥ 3 |  |

*Notes*

Hazard ratios (HRs) and 99% confidence intervals (CIs) were estimated by means of Cox proportional hazards models stratified by age group (30–49, 50–64, and 65–80 years)
Parity was categorized as 0, 1–2, and ≥3 children.
*Adjusted for age at the beginning of the study

**Adjusted for entry year in the age-based follow-up period

Models were adjusted for ethno-religious group, education, country of origin, locality size, and calendar year of entry into each age band

*Table 4:* Results *of multivariable Cox models for predicting breast cancer mortality rates by number of children and sociodemographic variables (hazard ratio)* *in the total population and age-based follow-up groups (30–49, 50–64, and 65–80 years)*

| ****Model D**  **65–80 bracket age-based follow-up group** | ****Model C**  **50–64 bracket age-based follow-up group** | **Model B****  **30–49 bracket age-based follow-up group** | ***Model A**  **total population** |  |  |
| --- | --- | --- | --- | --- | --- |
| **752,958** | **857,104** | **866,160** | **/** |  | **N** |
| **1.00** | **1.00** | **1.00** | **1.00** | **0** | Number of children |
| **1.237**  **(1.045–1.466)** | **1.071**  **(0.949–1.209)** | **1.656**  **(1.349–2.033)** | **1.216**  **(1.117–1.324)** | **1–2** |  |
| **0.989**  **(0.834–1.173)** | **0.935**  **(0.830–1.054)** | **1.551**  **(1.271–1.893)** | **1.056**  **(0.970–1.149)** | **≥ 3** |  |
| 1.00 | 1.00 | 1.00 | 1.00 | Non-Haredi Jewish | Ethno-religious group |
| 0.711  (0.54–0.935) | 0.873  (0.703–1.084) | 0.720  (0.623–0.865) | 0.776  (0.658–0.915) | Haredi Jewish |  |
| 0.825  (0.648–1.051) | 1.079 (0.922–1.263) | 0.784  (0.601–0.962) | 0.852  (0.715–0.911) | Arab and other |  |
| 1.00 | 1.00 | 1.00 | 1.00 | 0–8 | Education (years) |
| 1.478  (1.263–1.665) | 1.558  (1.392–1.745) | 1.254  (1/058–1/487) | 1.471  (1.361–1.589) | 9–12 |  |
| 1.422  (1.263–1.729) | 1.598  (1.428–1.788) | 0.965  (0.886–1.051) | 1.388  (1.283–1.501) | ≥ 13 |  |
| 1.00 | 1.00 | 1.00 | 1.00 | Asia/Africa | Country of origin |
| 0.977  (0.836–1.142) | 0.950  (0.855–1.055) | 1.028 (0.873–1.210) | 0.994  (0.923–1.072) | Israel |  |
| 1.019  (0.963–1.203) | 1.088  (0.974–1.215) | 1.254  (1.059–1.487) | 1.136  (1.050–1.230) | Europe/America |  |
| 1.00 | 1.00 | 1.00 | 1.00 | Small | Size of locality of residence |
| 1.278  (1.077–1.516) | 1.123  (1.007–1.251) | 1.298  (1.094–1.541) | 1.205  (1.113–1.304) | Large |  |

*Notes*

Model A represents a time-dependent Cox proportional hazards model in which each woman contributed person-time to all relevant age bands (30–49, 50–64, and 65–80 years) based on her age at each point in follow-up.
Models B, C, and D are age-stratified Cox models limited to specific age bands:

Model B: 30–49 years,
Model C: 50–64 years,
Model D: 65–80 years.

In these models, each woman contributed follow-up time only during the period she fell within the specified age range
* Model A: Adjusted for age at the beginning of study

**Models B, C, and D: Adjusted for entry year in the age-based follow-up period
