# Supplementary material for: The parity paradox: does number of children (parity) influence breast cancer mortality across the life course?
Source: BMC Cancer. 2025 Nov 11;25:1750. doi: 10.1186/s12885-025-14993-1 (PMC12606814; doi:10.1186/s12885-025-14993-1)
Supplement: Supplementary file 2 — Supplementary Material 2. [file 12885_2025_14993_MOESM2_ESM.docx]

*Figure 1: Analysis of breast cancer mortality rates by number of children, adjusted for age at the beginning of the follow-up period, level of education, ethno-religious group, country of origin, size of locality of residence in the total population, and age-based follow-up groups (30–49, 50–64, and 65–80 years)*

| Total study period | 30–49 bracket age-based study period |
| --- | --- |
| 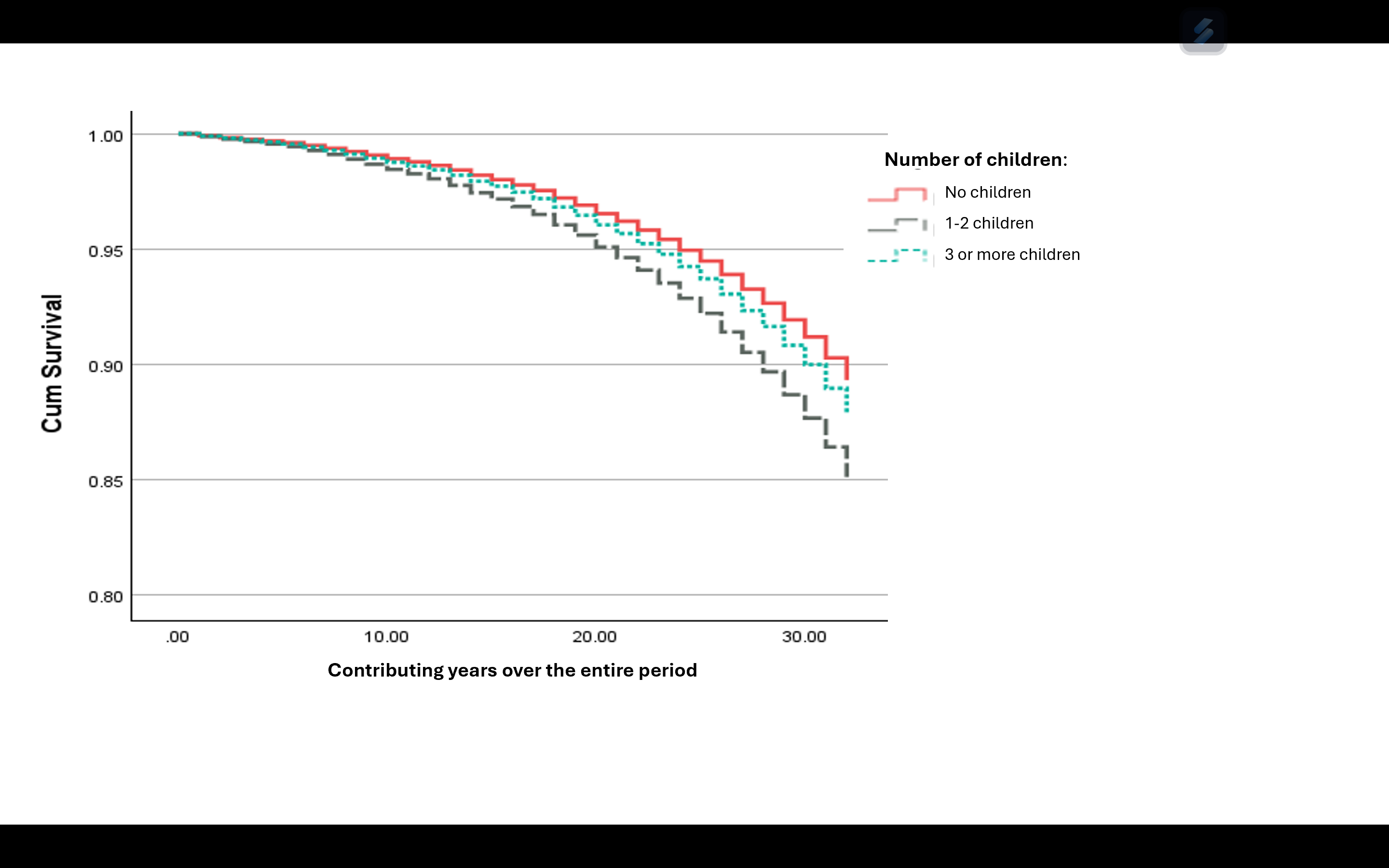 | 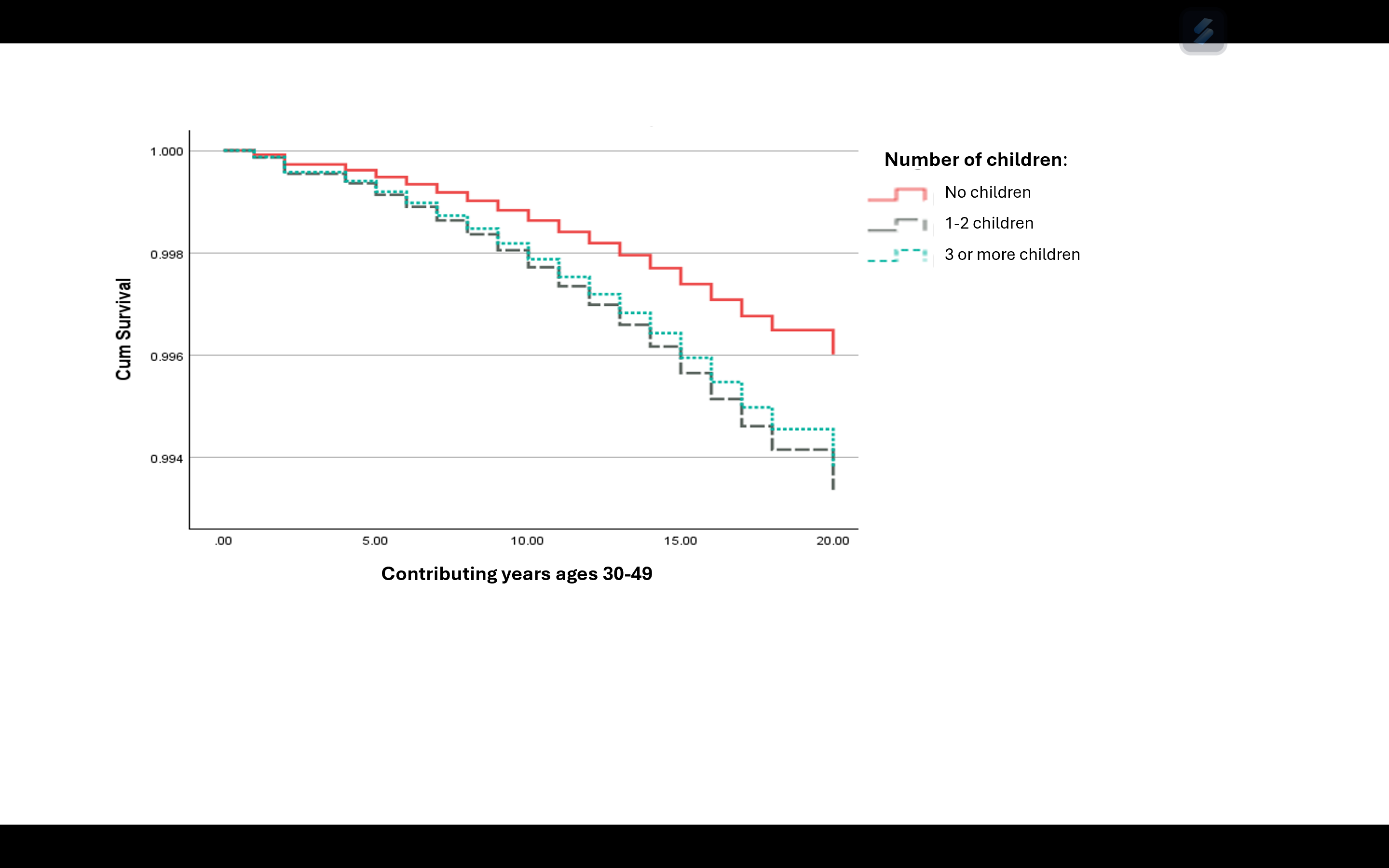 |
| 50–64 bracket age-based study period | 65–80 bracket age-based study period |
| 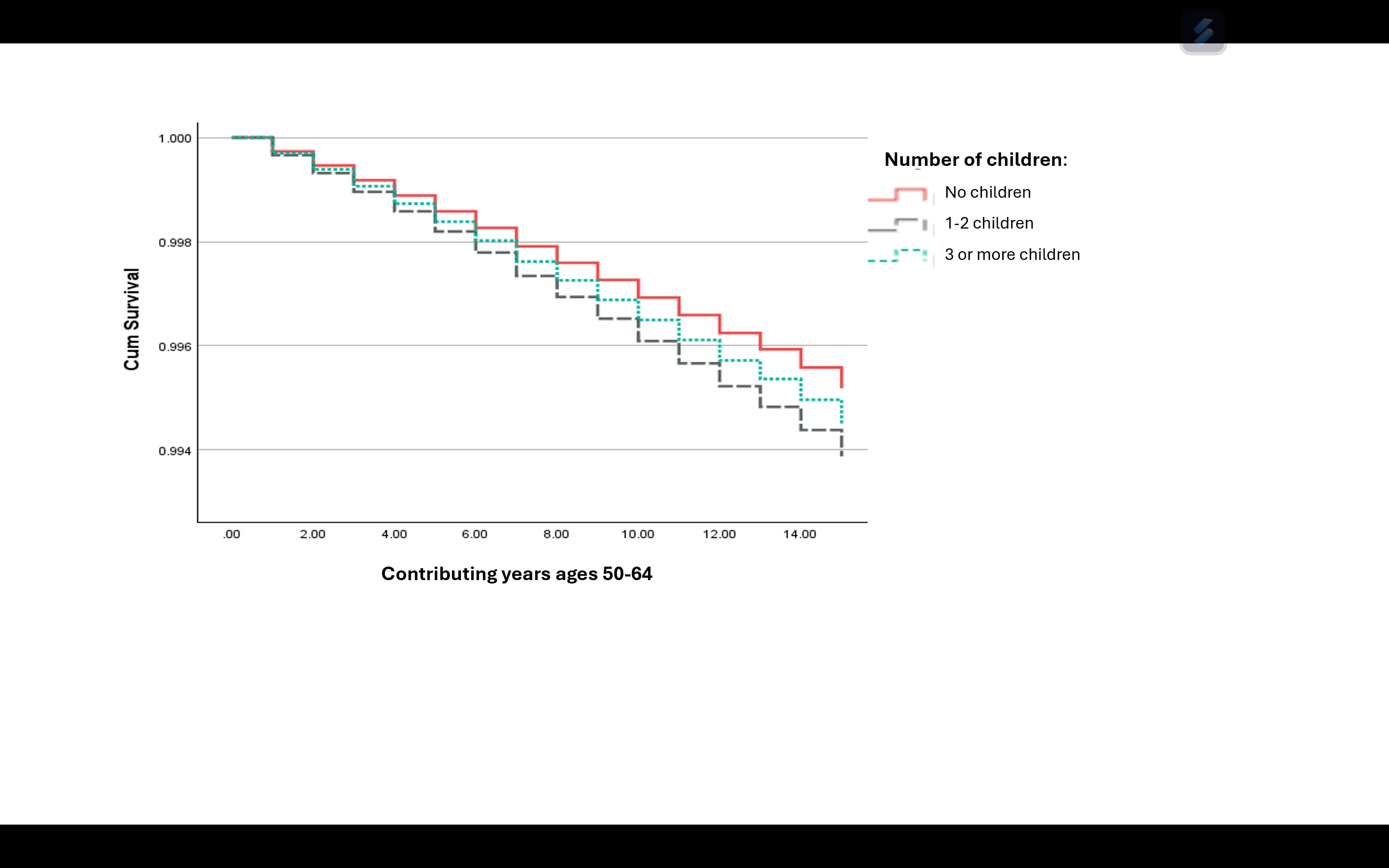 | 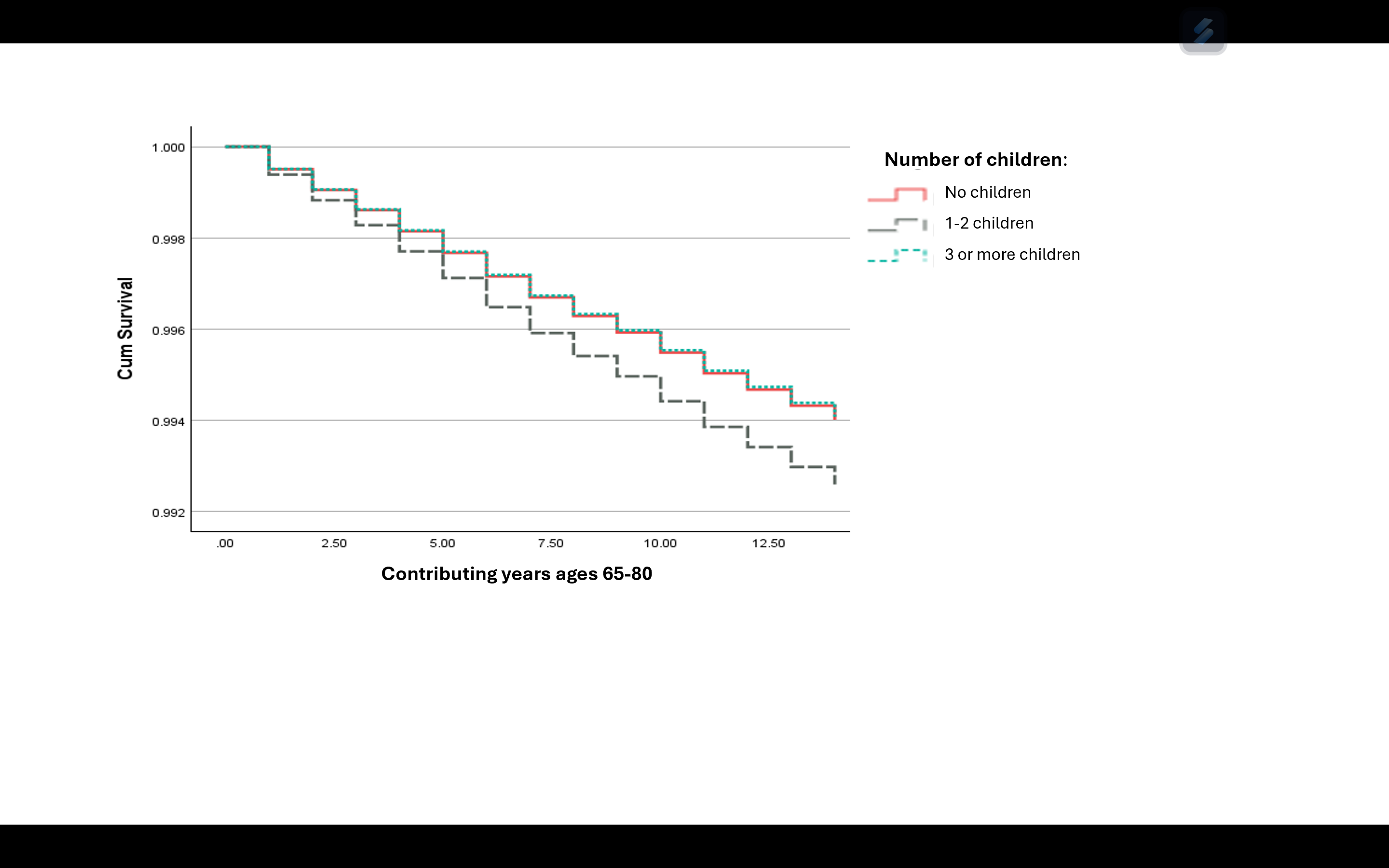 |

*Notes*

Hazard ratios (HRs) and 99% confidence intervals (CIs) were estimated by means of age-stratified Cox proportional hazards models
Women were grouped by age at follow-up: 30–49, 50–64, and 65–80 years
Parity was categorized as 0 (reference), 1–2, and ≥3 children
Models were adjusted for ethno-religious group, education, country of origin, locality size, and calendar year of entry in each age band
